# Trivalent chromium versus baricitinib for rheumatoid arthritis treatment: first phase 2/3 randomized controlled trial, is trivalent chromium the upcoming immune-modulator?

**DOI:** 10.1007/s10787-024-01515-x

**Published:** 2024-07-19

**Authors:** Sally S. Hassouna, Omneya Mohamed-Ayman Abdel-Moniem

**Affiliations:** 1 Internal Medicine Department, Rheumatology and Clinical Immunology Unit, Faculty of Medicine, Alexandria University, Alexandria, Egypt; 2 Rheumatology, Rehabilitation and Physical Medicine Department, Faculty of Medicine, Alexandria University, Alexandria, Egypt

**Keywords:** Rheumatoid arthritis, Trivalent chromium, Baricitinib, CDAI, SDAI, HAQDI, DAS28-CRP, DAS28-ESR, ACR response, GLOESS, SH, PDUS, Joints effusions, Low disease activity and disease remission

## Abstract

**Background:**

Rheumatoid arthritis (RA) is a debilitating disease mainly treated by DMARDs. Baricitinib is one of the emerging DMARDs with strong anti-rheumatic effects but has serious side effects. Trivalent chromium (Cr III) is a natural element with anti-inflammatory properties. Trivalent chromium (Cr III) is introduced for the first time to study its effect and safety in treatment of RA patients and compared to those of baricitinib.

**Methods:**

This is a phase 2/3 randomized controlled trial where RA patients were divided in a ratio of 2:1 according to the newly introduced medication either Cr (III) (group A) or baricitinib (group B). Patients attended three visits on day 0, after 3 weeks and 12 weeks, disease activity was scored. Hands ultrasound was done and reassessed. Side effects were monitored throughout the study.

**Results:**

DAS28-CRP improved by 26.9% and 11.8% on third visit for Cr III and baricitinib, respectively (*p* = 0.001). DAS28-ESR improved by 25.6% and 7.74% on third visit for Cr III and baricitinib, respectively (*p* =  < 0.001). ACR 50 was 18.8% for Cr III and 5.7% for baricitinib on second visit. ACR 70 was 25% for Cr III and 0% for baricitinib on third visit (*P* =  < 0.001). Ultrasound GLOESS, SH, PDUS, joints effusions improved by 38.9%, 38.4%, 56.7% and 74.8% for Cr III, while by 10.5%, 3.75%, 59.6% and worsening of joints effusions happened with baricitinib on third visit. *p* = 0.022 and 0.002 between groups for GLOESS and SH improvement, respectively.

**Conclusions:**

Cr III has shown very promising fast clinical and sonographic results in treating RA patients which were surprisingly superior to baricitinib in most aspects. Furthermore, Cr III is potentially safe with evidently fewer side effects than baricitinib and other DMARDs, however, long-term safety is still not established.

(IRB No.: 00012098- FWA No.: 00018699, Serial number: 040457) ClinicalTrials.gov ID: NCT05545020.

## Introduction

Rheumatoid arthritis (RA) is a known disease with an autoimmune pathogenesis. The disease mainly causes joints inflammation and may have extra-articular manifestations. (National Institute of Arthritis and Musculoskeletal and Skin Diseases (NIH) 2014), (Majithia and Geraci 2007).

Trying to delay disease progression, to hinder joints destruction and to decrease morbidities, several medications with different mechanisms of action are used for RA treatment. These medications include NSAIDs and steroids which are used for symptoms relief, synthetic Disease-modifying anti-rheumatic drugs (sDMARDs) which involves conventional sDMARDs e.g. methotrexate, leflunamide, salazopyrine, hydroxychloroquine and targeted sDMARDs i.e. Jannus kinase inhibitor (JAK inhibitors) and biologic DMARDs which are used to slow down the occurrence of disease consequences. (Singh et al. 2016) Meanwhile, these medications have multiple side effects most of them are deleterious to patients and may cause serious insults (Stuart 2023) which necessitates looking for alternatives that are safe and effective to treat the disease and halt its complications.

Trivalent chromium (Cr III) is a supplement used as a mediator of metabolism which increases insulin sensitivity, (EFSA Panel on Dietetic Products and Allergies 2014), (Hua et al. 2012) was reported to be deficient in some RA patients (Mohamed 2016) and has shown evidence of its regulatory roles on various immune pathways with anti-inflammatory properties, (Hassouna et al. 2022), (Moradi et al. 2019), (Jain et al. 2010), (Hoffman et al. 2014), (Kolahian et al. 2015), (Rhee et al. 2002), (Arunkumar et al. 2000) with anti-oxidant effects, (Cheng et al. 2004) was successful in treatment of RA disease model (Hassouna et al. 2022) and revealed to have protective properties; (Su et al. 2023), (Roczniak et al. 2018), (McCarty 1995) yet controversial; on bones (Ning et al. 2002), which makes Cr III an excellent candidate to experience for treatment of RA patients especially since Cr III is considered a human natural trace mineral which has beneficial outcomes on blood sugar and lipids including prevention of hyper-cholestrolemia. (EFSA Panel on Dietetic Products and Allergies 2014), (Hua et al. 2012), (Feng et al. 2018). Other Cr III effects that may be beneficial actions for RA patients include the possibility of its anti-depressive property (Komorowski et al. 2012) and lowering body weight in some subjects. (Anderson 1998).

Baricitinib is a reversible JAK inhibitor that inhibits JAK 1 with less inhibitory effects on tyrosine kinase 2 (JAK2) and least on JAK3 through acting on pathways involving STAT proteins, which in turn modulates expression of genes in immunological cells, for this reason it is used for treatment of RA and has shown good treatment results in the disease, (European Medicines Agency 2017) however, baricitinib causes several side effects that are sometimes life threatening. Some of those side effects are due to hypercoagulability and hypercholesterolemia provoked by the medication. (European Medicines Agency 2017).

This study is a challenge for Cr III versus a strong medication like baricitinib to compare their effectiveness in RA treatment and if Cr III as a natural product would be safe for patients to be a potential substitute or adjuvant for immune-modulatory medications in the near future.

## Methods

After acceptance of Ethics Committee, Faculty of Medicine, Alexandria University, Egypt for performing this trial (IRB No.: 00012098- FWA No.: 00018699, Serial number: 040457) and the study was registered in ClinicalTrials.gov with ID: NCT05545020, RA patients according to ACR 1987 And 2010 criteria (Arnett et al. 1988), (Aletaha et al. 2010) were recruited from Alexandria and surrounding governorates to join the study at Faculty of Medicine, Alexandria University, Egypt main hospital. Sample size was estimated by biomedical Informatics and Medical Statistics Department, Medical Research Institute, Faculty of Medicine, Alexandria University, Egypt, using NCSS 2004/PASS 2000 software. (Hintze 2000).

Patients with overlap syndrome, with other known cause of arthritis, hepatitis C virus, known hepatic or renal patients, known to have or prone to have hypoglycemia, history of malignancies and pregnant female patients were excluded from the study. Patients included in the study were divided into two groups: * Group A: (intervention group) which consisted of volunteer RA patients who accepted to take Cr III in the form of amino acid chelate with a dose of (3.5 ± 0.5 μg/kg bodyweight i.e. one tablet or two tablet daily or one tablet and two tablets on alternate days before breakfast or before breakfast and lunch) referring to the disease model study [7] but with a lower dose trying to avoid side effects while keeping efficacy by using the 200 μg undividable tablet and * Group B: (control group) consisted of patients that baricitinib was newly prescribed to them in a dose of 4 mg once daily. All patients in both groups were asked about their medications and patients who had any change (beginning or stoppage) in DMARDs through the 3 months before engagement in the study were excluded to assure no impact of other medications disease activity and treatment response during the study. (Cohen et al. 2024).

After selection of patients, they were informed about the study design and informed consent was obtained from all participating patients. Study was depending on attending three visits with the beginning of the studied medication (Cr III in intervention group and baricitinib in control group): first visit on Day 0, second visit on day 21 and third visit on week 12 to give a chance period for both studied medications to show their effects, giving that most of DMARDs take that period to give their full action. (Cohen et al. 2024).

During each visit, patients were thoroughly examined; 28 tender joint count (TJC) and 28 swollen joint count (SJC) (Grunke et al. 2012) (28 TJC and 28 SJC include: both shoulders, both elbows, both knees, both wrists, metacarpophalangeal joints and proximal interphalangeal joints) were assessed and recorded. Physician Global Assessment score (PGA), Patient global Assessment score (PtGA), and joint pain according to Visual analogue scale were recorded each visit. Health Assessment Questionnaire Disability index (HAQ-DI) was also assessed and recorded for every on each visit. (Kumar et al. 2017), (Aletaha and Smolen 2005).

Lab investigations: inflammatory markers (erythrocyte sedimentation rate (ESR) and C reactive protein (CRP)) were measured each visit. Blood count were done for all patients and investigations relevant to screen toxicity in intervention group e.g., liver enzymes (SGOT and SGPT), some renal functions investigations (serum urea and serum creatinine) were assessed at the beginning and at the end of the study. Random blood sugar was measured through the study for intervention group. Disease activity scores were calculated each visit including: Clinical disease activity score (CDAI), Simplified disease activity score (SDAI), Disease Activity Score in 28 joints calculated with CRP (DAS28-CRP), Disease activity score in 28 joints calculated with ESR (DAS28-ESR) and American College of Rheumatology Score (ACR). (Felson et al. 1995).

Ultrasound examination of hands joints was done at the beginning and at the end of the study and Global OMERACT-EULAR Synovitis Score (GLOESS), synovial hypertrophy (SH), power Doppler/greyscale ultrasound (PDUS) and number of effusions were estimated for selected patients. (Naredo et al. 2011).

## Statistical analysis

Data were analyzed through using (IBM SPSS) software package; version 20.0. [Armonk, NY: IBM Corp]. Categorical data were in the form of numbers and percentages. [Chi-square test] was used to compare between groups. When > 20% of cells have less than 5- expected count. [Fisher Exact or Monte Carlo correction test] was applied. [Shapiro–Wilk test] was used for testing normality of continuous data. Quantitative data were expressed in the form of (minimum and maximum), mean with standard deviation and median. Comparison of the quantitative normally distributed variables between groups was done using [Student t-test]. While [ANOVA test] with repeated measures was applied to compare between groups, while, the test used for pairwise comparisons was [Post Hoc test (Bonferroni adjusted)]. For quantitative variables which are abnormally distributed [Mann Whitney test] was used to compare groups. On the other hand, [Wilcoxon signed ranks test] was applied for comparison between two periods, while [Friedman test] was used for comparison between visits and for pairwise comparisons [Post Hoc Test (Dunn's)] was used. judged Judgement level of significance of results was at the 5%.

## Results

Over hundred RA patients were screened searching for eligible patients to roll in the study from December 2022 to January 2024. Sixty nine RA patients participated in the study with a ratio 1:2 for Cr III and baricitinib, respectively, with 9 drop outs (3 and 6 patients from intervention group and from control group, respectively), 5 patients (12.5%) stopped baricitinib for side effects and there was one death (2.5%) documented in baricitinib group, while there were two patients (10%) who stopped Cr III supplement after two to three doses of a tablet per day dose, another patient (5%) stopped Cr III after 1 month with improvement of disease activity due to complaint of headache and non-preference of body weight loss and only one patient (5%) was recommended to stop the Cr III supplement after 1 week due to complaint of mild facial swelling and disease activity improvement was evident after only 1 week of treatment. Side effects that appeared in both groups are listed in (Table 1).

**Table 1:** (A) Different parameters during each visit and difference for each group on different visits, (B): showing disease activity scores and their differences on different visits, (C) showing hands ultrasound scores on the first and the third visits

	Trivalent chromium (*n* = 16)						Baricitinib (*n* = 35)					
	Day 0	3 weeks	12 weeks	Test of Significance (*p*)	Significance between visits		Day 0	3 weeks	12 weeks	Test of Significance (*p*)		Significance between visits
(A) Different parameters during each visit and difference for each group on different visits												
Morning stiffness												
Mean ± SD	25.5 ± 20	15.5 ± 15.1	15 ± 20	Fr = 10.739^*^	*p*_1_ = 0.018^*^		37.9 ±	26.6 ± 34.5	31.2 ± 35.1	Fr = 22.738^*^ (< 0.001^*^)		*p*_1_ < 0.001*
				(0.005^*^)	*p*_2_ = 0.006^*^		39.6					*p*_2_ = 0.001^*^,
Median (Min.–Max.)	15 (5–60)	13.5 (0 –60)	3.75 (0 –60)		*p*_3_ = 0.70		30 (0–210)	19 (0–180)	29 (0–180)			*p*_3_ = 0.339
SJC 28												
Mean ± SD	6.31 ± 4.53	3.94 ± 3.17	1.63 ± 1.09	Fr = 13.236^*^	*p*_1_ = 0.052		2.29 ± 1.90	1.60 ± 1.31	1.26 ± 1.38	Fr = 17.413^*^ (< 0.001^*^)		*p*_1_ = 0.023^*^,
				(0.001^*^)	*p*_2_ = 0.001^*^							*p*_2_ < 0.001^*^,
Median (Min.–Max.)	6 (1–13)	3 (0–10)	1.50 (0–4)		*p*_3_ = 0.157		2 (0–8)	1 (0–6)	1 (0–6)			*p*_3_ = 0.169
TJC 28												
Mean ± SD	13 ± 8.41	8.63 ± 6.60	6.50 ± 5.80	Fr = 20.548^*^	*p*_1_ = 0.006^*^		15.7 ± 7.14	12.5 ± 7.18	13.5 ± 6.93	Fr = 15.679^*^ (< 0.001^*^)		*p*_1_ < 0.001^*^,
				(< 0.001^*^)	*p*_2_ < 0.001^*^							*p*_2_ = 0.036^*^,
Median (Min.–Max.)	13 (2–27)	8 (0–23)	6 (0–19)		*p*_3_ = 0.093		16 (1–28)	13 (1–28)	13 (2–28)			*p*_3_ = 0.083
Physician global assessment scale score					*p*_1_ = 0.013^*^							*p*_1_ < 0.001^*^,
Mean ± SD	5.69 ± 1.96	3.38 ± 2.09	2 ± 1.75	Fr = 26.133^*^	*p*_2_ < 0.001^*^		5.69 ± 1.66	4.26 ± 1.70	3.94 ± 1.47	Fr = 36.358^*^ (< 0.001^*^)		*p*_2_ < 0.001^*^,
Median (Min.–Max.)	5 (3–10)	3 (1–9)	2 (0–6)	(< 0.001^*^)	*p*_3_ = 0.013^*^		6 (1–9)	4 (1–8)	4 (1–7)			*p*_3_ = 0.403
Patient global assessment scale score												
Mean ± SD	5.78 ± 2.20	4.59 ± 2.20	3.31 ± 2.55	Fr = 12.035^*^	*p*_1_ = 0.077		7.49 ± 1.76	6.53 ± 2.16	5.30 ± 1.98	Fr = 33.294^*^ (< 0.001^*^)		*p*_1_ = 0.006^*^,
				(0.002^*^)	*p*_2_ = 0.001^*^							*p*_2_ < 0.001^*^,
Median (Min.–Max.)	5.50 (1–9)	4.50 (1 –9)	3 (0–10)		*p*_3_ = 0.133		8 (4–10)	7 (1–10)	5 (1–9)			*p*_3_ = 0.010^*^
Visual pain analogue scale score												
Mean ± SD	8.50 ± 3.35	6 ± 1.70	4.22 ± 2.21	Fr = 23.310^*^	*p*_1_ = 0.022^*^		8.01 ± 1.51	6.89 ± 1.79	5.42 ± 2	Fr = 43.736^*^ (< 0.001^*^)		*p*_1_ = 0.005^*^,
				(< 0.001^*^)	*p*_2_ < 0.001^*^							*p*_2_ < 0.001^*^,
Median (Min.–Max.)	8 (5–20)	5.5 (4–9.5)	4 (0–8)		*p*_3_ = 0.022^*^		8 (4–10)	7 (2–10)	5 (1.80–9)			*p*_3_ < 0.001^*^
ESR (mm/hr)												
Mean ± SD	23.4 ± 12.9	24 ± 13.4	20.2 ± 11.2	Fr = 2.100	–		23.4 ± 12.9	24 ± 13.4	20.2 ± 11.2	Fr = 3.481		–
Median (Min.–Max.)	22.3 (8–59)	24 (4–56)	16.9 (6–42)	-0.35			22.3 (8–59)	24 (4–56)	16.9 (6–42)	-0.175		
CRP (mg/L)												
Mean ± SD	10.7 ± 10.7	8.78 ± 7.01	11.2 ± 13.1	Fr = 1.220	–		10.7 ± 10.7	8.78 ± 7.01	11.2 ± 13.1	Fr = 11.529^*^ (0.003^*^)		*p*_1_ = 0.001^*^,
				-0.543								*p*_2_ = 0.094,
Median (Min.–Max.)	6.95 (2.40–38.9)	6.24 (2.3–27.2)	7.55 (1.70–54.2)				6.95 (2.40–38.9)	6.24 (2.3–27.2)	7.55 (1.70–54.2)			*p*_3_ = 0.094
HAQ-DI					*p*_1_ = 0.112							*p*_1_ < 0.001^*^
Mean ± SD	1.55 ± 0.59	1.26 ± 0.58	0.80 ± 0.59	Fr = 21.934^*^ (< 0.001^*^)	*p*_2_ < 0.001^*^		2.16 ± 0.41	1.68 ± 0.58	1.54 ± 0.61	Fr = 35.368^*^ (< 0.001^*^)		*p*_2_ < 0.001^*^
Median (Min.–Max.)	1.50 (0.50–2.50)	1.0 (0.50–2.25)	0.57 (0.13–2.13)		*p*_3_ = 0.004^*^		2.13 (1.13–3.0)	1.75 (0.50–2.75)	1.71 (0.25–2.63)			*p*_3_ = 0.031^*^
Mild to moderate (0 – < 1)	2 (12.5%)	7 (43.8%)	12 (75.0%)				0 (0.0%)	5 (14.3%)	6 (17.1%)			
Moderate to severe (1 – < 2)	9 (56.3%)	6 (37.5%)	3 (18.8%)				8 (22.9%)	17 (48.6%)	21 (60.0%)			
Severe to very severe (2 – < 3)	5 (31.3%)	3 (18.8%)	1 (6.3%)				26 (74.3%)	13 (37.1%)	8 (22.9%)			
Completely disabled (3)	0 (0.0%)	0 (0.0%)	0 (0.0%)				1 (2.9%)	0 (0.0%)	0 (0.0%)			
(B): showing disease activity scores and their differences on different visits												
DAS28-CRP												
Disease remission	0 (0.0%)	0 (0.0%)	2 (12.5%)		Fr = 18.667^*^	*p*_1_ = 0.034^*^	0 (0.0%)	2 (5.7%)	0 (0.0%)	Fr = 20.269^*^ (< 0.001^*^)	*p*_1_ = 0.010^*^,	
Low disease activity	0 (0.0%)	2 (12.5%)	4 (25.0%)		(< 0.001^*^)	*p*_2_ = 0.001^*^	1 (2.9%)	2 (5.7%)	4 (11.4%)		*p*_2_ = 0.005^*^,	
Moderate disease activity	8 (50.0%)	12 (75.0%)	8 (50.0%)			*p*_3_ = 0.289	12 (34.3%)	21 (60.0%)	22 (62.9%)		*p*_3_ = 0.811	
High disease activity	8 (50.0%)	2 (12.5%)	2 (12.5%)				22 (62.9%)	10 (28.6%)	9 (25.7%)			
Mean ± SD	5.08 ± 1.06	4.31 ± 0.97	3.71 ± 1.11		F = 41.391^*^	*p*_1_ < 0.001^*^	5.17 ± 0.80	4.63 ± 1	4.56 ± 0.89	F = 14.988^*^ (< 0.001^*^)	*p*_1_ = 0.001^*^,	
					(< 0.001^*^)	*p*_2_ < 0.001^*^					*p*_2_ < 0.001^*^,	
Median (Min.–Max.)	5.15 (3.53–7.12)	4.13 (3.05–6.70)	3.66 (1.87–6.25)			*p*_3_ = 0.002^*^	5.24 (2.78–6.44)	4.75 (1.93–6.12)	4.66 (2.76–6.20)		*p*_3_ = 1.000	
DAS28-ESR												
Disease remission	0 (0.0%)	0 (0.0%)	1 (6.3%)		Fr = 9.500^*^	*p*_1_ = 0.251	0 (0.0%)	1 (2.9%)	0 (0.0%)	Fr = 5.892 (0.053)	–	
Low disease activity	0 (0.0%)	1 (6.3%)	1 (6.3%)		(0.009^*^)	*p*_2_ = 0.042^*^	1 (2.9%)	0 (0.0%)	1 (2.9%)			
Moderate disease activity	7 (43.8%)	9 (56.3%)	11 (68.8%)			*p*_3_ = 0.377	6 (17.1%)	11 (31.4%)	12 (34.3%)			
High disease activity	9 (56.3%)	6 (37.5%)	3 (18.8%)				28 (80.0%)	23 (65.7%)	22 (62.9%)			
Mean ± SD	5.44 ± 1.14	4.71 ± 1.02	4.02 ± 0.88		F = 32.391^*^	*p*_1_ = 0.004^*^	5.71 ± 0.87	5.29 ± 1.07	5.26 ± 0.99	F = 7.714^*^ (0.002^*^)	*p*_1_ = 0.006^*^,	
					(< 0.001^*^)	*p*_2_ < 0.001^*^					*p*_2_ < 0.001^*^,	
Median (Min.–Max.)	5.73 (3.33–7.26)	4.87 (3.09–6.63)	4 (2.47–5.36)			*p*_3_ = 0.002^*^	5.81 (3–7)	5.37 (1.95–6.92)	5.33 (3.03–6.96)		*p*_3_ = 1.000	
CDAI						*p*_1_ = 0.027^*^					*p*_1_ < 0.001^*^,	
Mean ± SD	30.9 ± 13.2	20.5 ± 10.2	13.4 ± 9.26		Fr = 22.317^*^	p_2_ < 0.001^*^	31.2 ± 9.50	24.9 ± 10.3	24 ± 9.42	Fr = 30.507^*^ (< 0.001^*^)	*p*_2_ < 0.001^*^,	
Median (Min.–Max.)	30.5(12–52.5)	18(7–49.5)	12.0(0–32)		(< 0.001^*^)	*p*_3_ = 0.013^*^	32 (7–49)	25.7 (4–45)	25.3 (7–41)		*p*_3_ = 0.120	
SDAI						*p*_1_ = 0.013^*^					*p*_1_ < 0.001^*^,	
Mean ± SD	24.4 ± 12.5	16.9 ± 8.45	11.9 ± 6.75		Fr = 19.625^*^	*p*_2_ < 0.001^*^	23.2 ± 8.73	18.7 ± 8.64	19.3 ± 8.21	Fr = 19.943^*^ (< 0.001^*^)	*p*_2_ = 0.006^*^,	
Median (Min.–Max.)	24.1 (7.53–45.3)	14.6 (6.16–39.7)	10.9 (1.87–25.1)		(< 0.001^*^)	*p*_3_ = 0.052	23.6 (3.78–39.4)	19.3 (3.09–37.1)	20 (4.76–34.74)		*p*_3_ = 0.094	
ACR score						*p*_1_ = 0.001^*^					*p*_1_ < 0.001^*^	
						*p*_2_ < 0.001^*^					*p*_2_ < 0.001^*^	
Mean ± SD	35.1 ± 13.6	23.2 ± 10	15.1 ± 8.72		F = 35.753^*^	*p*_3_ < 0.001^*^	33.9 ± 9.50	26.9 ± 10	25.6 ± 9.71	F = 23.870^*^	*p*_3_ = 1.000	
					(< 0.001^*^)					(< 0.001^*^)		
(C) showing hands ultrasound scores on the first and the third visits												
GLOESS						p_0_ = 0.005^*^						p_0=_ 0.133
Mean ± SD	36.1 ± 23.1		23.6 ± 16.3		Z = 2.807^*^		12.8 ± 9.1		10.4 ± 5.4	Z = 1.501		
Median (Min.–Max.)	49.5 (3–56)		27.5 (1–45)				9 (3–34)		10 (1–21)			
SH						p_0=_ 0.005^*^						p_0=_ 0.424
Mean ± SD	35.9 ± 23.2		23.4 ± 16.3		Z = 2.814^*^		12 ± 9.2		10 ± 5.5	Z = 0.800		
Median (Min.–Max.)	49.5 (3–56)		27.5 (1–45)				9 (0–34)		10 (0–21)			
PDUS						p_0=_ 0.102						p_0=_ 0.004^*^
Mean ± SD	2 ± 2.11		1.2 ± 2.15		Z = 1.633		3.8 ± 4.7		1.4 ± 1.79	Z = 2.870^*^		
Median (Min.–Max.)	1.5 (0–5)		0 (0–6)				3 (0–20)		0.5 (0–5)			
Effusions						p_0=_ 0.018^*^						p_0=_ 0.498
Mean ± SD	9 ± 7.44		1.6 ± 1.78		Z = 2.366^*^		0.95 ± 1.79		0.65 ± 1.69	Z = 0.677		
Median (Min.–Max.)	10.5 (0–19)		1 (0–4)				0 (0–7)		0 (0–7)			

*SD* standard deviation *Fr* friedman test, Sig. bet. periods was done using Post Hoc Test (Dunn's)

*F* f test (ANOVA) with repeated measures, Sig. bet. periods was done using Post Hoc Test (Bonferroni)

*U* mann whitney test*Z* wilcoxon signed ranks test

p: p value for comparing between the three studied periods

p_1_: p value for comparing between Day 0 and 3 weeks

p_2_: p value for comparing between Day 0 and 12 weeks

p_3_: p value for comparing between 3 and 12 weeks

p_0_: p value for comparing between Day 0 and 12 weeks

*: Statistically significant at *p* ≤ 0.05

## Patients’ characteristics

Patients were females except for one male patient in each group (5% for group A and 2.5% for group B) and were seropositive except for six patients (three patients in each group were seronegative (15% in group A and 7.5% in group B). 30% of all patients were having positive family history of the disease. Patients’ age was insignificantly different between both groups, *p* = 0.103 (Mean ± SD. = 42.9 ± 6 years old in group A with median = 42.5 (34–56) and Mean ± SD. = 46.35 ± 10.07 years old with median = 45 (33–71) in group B). Mean ± SD. of disease duration according to patients’ histories was 7.7 ± 5.1 years with median = 6 (0.5–24) in group A, while mean ± SD. was = 10.1 ± 8 years and median = 8 (0.6–40) in group B with no significant difference between both groups *p* = 0.375. Conventional DMARDs medications already taken by patients were methotrexate: (60%) in group A and (30%) in group B, leflunamide: (50%) in group A and (45%) in group B, salazopyrine: (15%) in group A and (15%) in group B and hydroxychloroquine: (55%) in group A and (35%) in group B. Corticosteroids and NSAIDs were taken by some patients in both groups with sometimes changing their doses. 20% of Cr III group and 20% of baricitinib group were on no treatment before engagement into the study where they only have taken Cr III in group A or baricitinib in group B.

## Efficacy of introduced medications

### A. Clinical examination and inflammatory makers

**Fig. 1:**
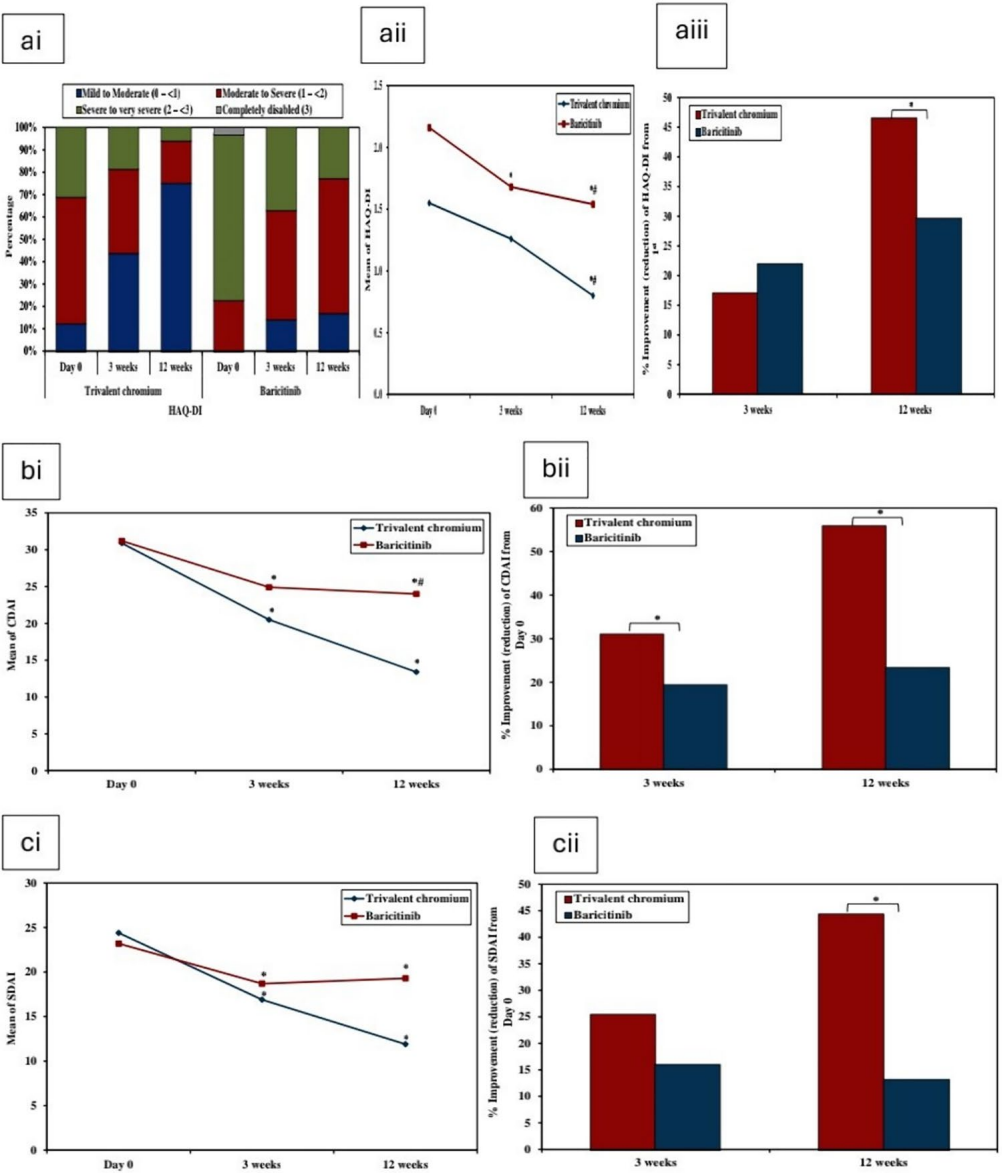
**ai** Grades of HAQ-DI for both groups on different visits, **aii** HAQ-DI values for Cr III and baricitinib across time. **aiii** Improvement of HAQ-DI in Cr III and baricitinib on the second and third visit. *: Significantly different from Day 0 and #: Significantly different from 3 weeks. **bi** CDAI values for Cr III and baricitinib across time **bii** Improvement of CDAI in Cr III and baricitinib on the second and third visit. **c**i SDAI values for Cr III and baricitinib across time **cii** Improvement of SDAI in Cr III and baricitinib on the second and third visit

Morning stiffness, clinical examination (TJC, SJC and PGA), PtGA, visual analogue scored joint pain, HAD-DI values in both groups and inflammatory markers (ESR and CRP) results and their differences on subsequent visits are shown in Table 1, HAQ-DI values and its improvement are shown on Table 1 and Fig. 1. Differences between groups for the previous items on different visits are added to the supplementary file. It should be mentioned that symptoms relief have begun in patients who received Cr III on the first days within the first week of its intake.

Some patients of whom represented with extraarticular manifestations (i.e., rheumatoid nodules) or secondary syndromes (i.e., eye and/or mouth dryness (secondary Sjogren’s syndrome) and fibromyalgia) experienced improvement of symptoms in both groups except for a few patients in baricitinib group who experienced exaggeration of secondary Sjogren’s syndrome.

### B. Disease activity scores

No significant differences were detected between groups in CDAI, SDAI, DAS28-CRP, DAS28-ESR and ACR score during first visit, while on the second visit there was a significant difference in CDAI between groups *p* = 0.042 and on the third visit there were significant differences between both groups (*p* = 0.002, 0.007, 0.005, < 0.001 and < 0.001 for CDAI, SDAI, DAS28-CRP, DAS28-ESR and ACR score between both groups, respectively.

**Fig. 2:**
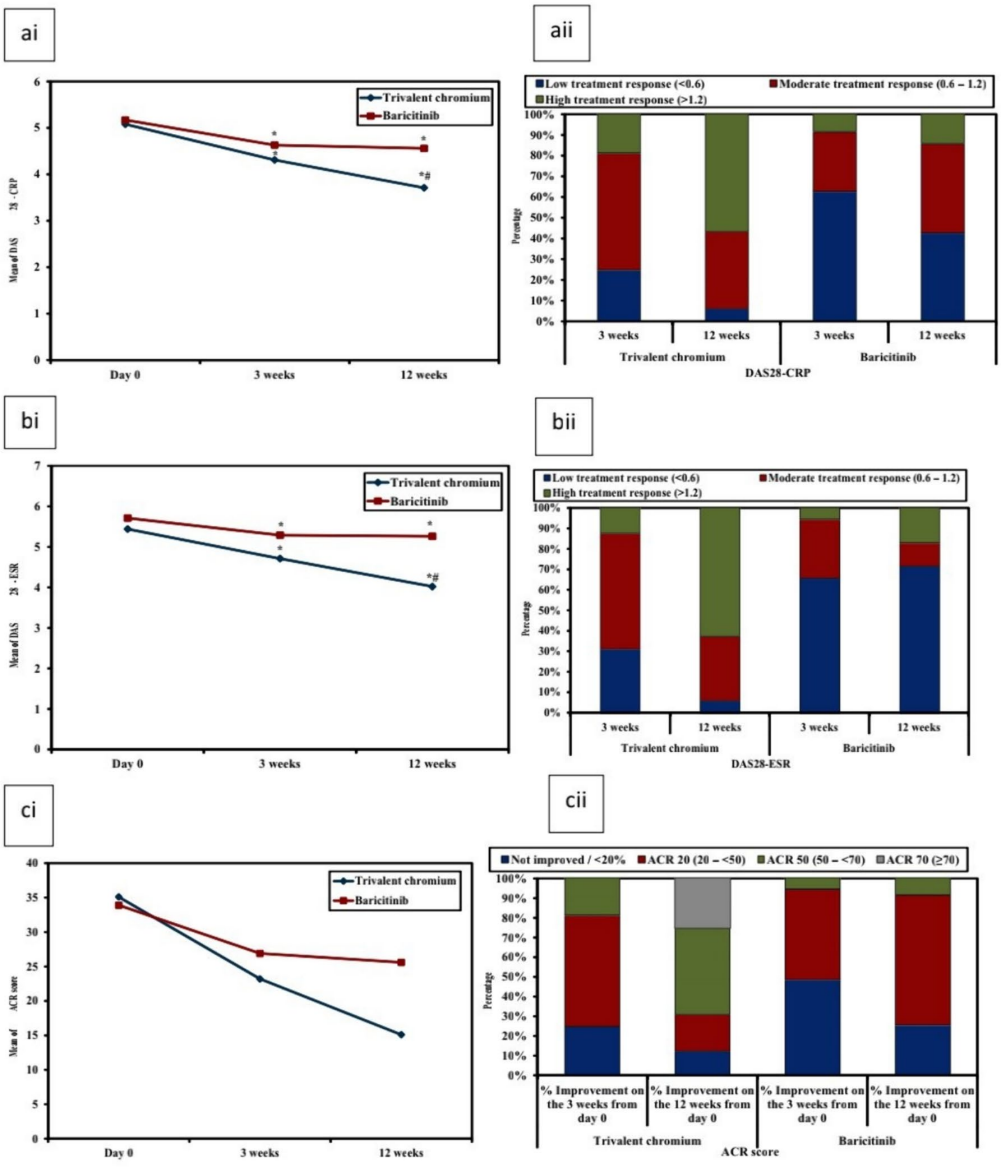
**ai** DAS28-CRP values for Cr III and baricitinib across time **aii** grades of DAS28-CRP for Cr III and baricitinib on different visits. **aiii** Improvement of DAS28-CRP in Cr III and baricitinib and grades of treatment response on the second and third visit. **bi** DAS28-ESR values for Cr III and baricitinib across time. **bii** Grades of DAS28-ESR for Cr III and baricitinib on different visits. **biii** improvement of DAS28-ESR in Cr III and baricitinib and grades of treatment response on the second and third visit. **ci** ACR scores of Cr III and baricitinib across time, **cii** ACR20, ACR50 and ACR70 of Cr III and baricitinib on the second and third visits. *: Significantly different from Day 0 and #: Significantly different from 3 weeks

Disease activity scores, differences between values on different visits, disease improvement and treatment response results for both groups are shown in Tables 1 and 2, Figs. 1, 2.

**Table 2:** (A) Comparison between the two studied groups according to disease activity scores (% Improvement), (B) Comparison between the two studied groups according to DAS28-CRP and DAS28-ESR (response to treatment), (C) showing improvement of hands ultrasound scores on the third visit

% Improvement (reduction) from Day 0	Trivalent chromium (*n* = 16)	Baricitinib (*n* = 35)	*U*	*P*
DAS28− CRP				
3 weeks				
Mean ± SD	14.9 ± 8.87	10.4 ± 15.4	192.50	0.076
Median (Min.–Max.)	16.2 (− 0.56–29.4)	10.3 (− 21.9–63.5)		
12 weeks				
Mean ± SD	26.9 ± 15.2	11.8 ± 10.7	110.00^*^	0.001^*^
Median (Min.–Max.)	27.8 (− 5.67–60)	11.6 (− 8.19–37.5)		
DAS28− ESR				
3 weeks				
Mean ± SD	12.7 ± 12	7.40 ± 13.5	177.00^*^	0.037^*^
Median (Min.–Max.)	13.5 (− 11.9–39.5)	7.12 (− 23.1–49.9)		
12 weeks				
Mean ± SD	25.6 ± 10.7	7.74 ± 11.6	76.00^*^	< 0.001^*^
Median (Min.–Max.)	26 (2.72–44)	7.64 (− 11.3–37.1)		
CDAI				
3 weeks				
Mean ± SD	31.1 ± 22.3	19.3 ± 25.8	180.50^*^	0.043^*^
Median (Min.–Max.)	37.8 (− 25–56)	19.4 (− 49.1–90.2)		
12 weeks				
Mean ± SD	55.9 ± 30.6	23.2 ± 18.8	86.500^*^	> 0.001^*^
Median (Min.–Max.)	58.6 (− 25–100)	22.5 (− 11.1–65.2)		
SDAI				
3 weeks				
Mean ± SD	25.5 ± 25.2	16 ± 34.9	226.00	0.273
Median (Min.–Max.)	30.9 (− 39.4–59.7)	18.9 (− 93.9–88.2)		
12 weeks				
Mean ± SD	44.3 ± 42.3	13.2 ± 28	104.0^*^	> 0.001^*^
Median (Min.–Max.)	50.7 (− 95.2–88.1)	16.1 (− 56.1–72.9)		
HAQ–DI				
% Improvement (reduction) from 1st				
3 weeks				
Mean ± SD	17.1 ± 22	21.9 ± 24.1	*U* = 258.00	0.655
Median (Min.–Max.)	21.7 (− 30.4–56)	18.9 (− 19–76.5)		
12 weeks				
Mean ± SD	46.5 ± 38	29.5 ± 24.2	*U* = 151.50^*^	0.009^*^
Median (Min.–Max.)	54.9 (− 70.4–84.8)	25 (− 12.5–86.7)		
ACR score				
% Improvement from day 0				
3 weeks				
Not improved / < 20%	4 (25.0%)	17 (48.6%)	*χ*^2^ = 3.599	^MC^p = 0.142
ACR 20 (20 – < 50)	9 (56.3%)	16 (45.7%)		
ACR 50 (50 – < 70)	3 (18.8%)	2 (5.7%)		
ACR 70 (≥ 70)	0 (0.0%)	0 (0.0%)		
Mean ± SD	31 ± 20	20.2 ± 21.7	*U* = 189.00	0.065
Median (Min.–Max.)	34.6 (− 13–55.6)	20.1 (− 30.5–68.6)		
12 weeks				
Not improved / < 20%	2 (12.5%)	9 (25.7%)	*χ*^2^ = 19.567^*^	^MC^p > 0.001^*^
ACR 20 (20 – < 50)	3 (18.8%)	23 (65.7%)		
ACR 50 (50 – < 70)	7 (43.8%)	3 (8.6%)		
ACR 70 (≥ 70)	4 (25.0%)	0 (0.0%)		
Mean ± SD	53.7 ± 30.1	25.2 ± 16.3	*U* = 89.00^*^	> 0.001^*^
Median (Min.–Max.)	55.9 (− 32.8–98.6)	23.15 (− 7.86–62.7)		

Difference from first visit	Trivalent chromium (*n* = 16)	Baricitinib(*n* = 35)	χ^2^	*P*
DAS28-CRP				
3 weeks				
Low treatment response (< 0.6)	4 (25%)	22 (62.9%)	6.453^*^	^MC^p = 0.040^*^
Moderate treatment response (0.6–1.2)	9 (56.3%)	10 (28.6%)		
High treatment response (> 1.2)	3 (18.8%)	3 (8.6%)		
12 weeks				
Low treatment response (< 0.6)	1 (6.3%)	15 (42.9%)	11.811^*^	0.003^*^
Moderate treatment response (0.6–1.2)	6 (37.5%)	15 (42.9%)		
High treatment response (> 1.2)	9 (56.3%)	5 (14.3%)		
DAS28-ESR				
3 weeks				
Low treatment response (< 0.6)	5 (31.3%)	23 (65.7%)	5.426	^MC^p = 0.057
Moderate treatment response (0.6–1.2)	9 (56.3%)	10 (28.6%)		
High treatment response (> 1.2)	2 (12.5%)	2 (5.7%)		
12 weeks				
Low treatment response (< 0.6)	1 (6.3%)	25 (71.4%)	18.795^*^	< 0.001^*^
Moderate treatment response (0.6 – 1.2)	5 (31.3%)	4 (11.4%)		
High treatment response (> 1.2)	10 (62.5%)	6 (17.1%)		

	Trivalent chromium (*n* = 10)	Baricitinib (*n* = 20)	*U*	*P*
GLOESS				
% Improvement (reduction)				
Mean ± SD	38.9 ± 16.5	10.5 ± 32.5	48.500^*^	0.022^*^
Median (Min. – Max.)	37 (19.6 – 66.7)	5.56 (-50 –66.7)		
SH index				
% Improvement (reduction)	(*n* = 10)	(*n* = 19)		
Mean ± SD	38.4 ± 15.2	3.75 ± 29.3	31.00^*^	0.002^*^
Median (Min. – Max.)	39 (19.6 – 66.7)	0 (-42.9 – 63.6)		
PD index				
% Improvement (reduction)	(*n* = 6)	(*n* = 14)	41.00	0.968
Mean ± SD	56.7 ± 54.3	59.6 ± 48.6		
Median (Min.–Max.)	80 (− 20–100)	73.3 (-66.7–100)		
Day 0				
EFFUSIONS				
% Improvement (reduction)	(*n* = 7)	(*n* = 7)		
Mean ± SD	74.8 ± 22.3	-32.7 ± 254.2	21.00	0.710
Median (Min.–Max.)	72.7 (40–100)	71.4 (− 600–100)		
Median (Min.–Max.)	0 (− 100–100)	0 (− 25–100)		

*SD* standard deviation*U* mann whitney test*Z *wilcoxon signed ranks test

p: p value for comparing between the two studied groups

p_0_: p value for comparing between Day 0 and 12 weeks

*: Statistically significant at *p* ≤ 0.05

*SD* standard deviation*U* mann Whitney testχ^2^: Chi square test

*MC* monte Carlo*t* student t-test

p: p value for comparing between the two studied groups

*: Statistically significant at *p* ≤ 0.05

### C. Disease improvement and treatment response

Disease improvement and treatment response according to disease activity scores are shown on Table 2 and Figs. 1 and 2

### D. Ultrasound scoring

**Fig. 3:**
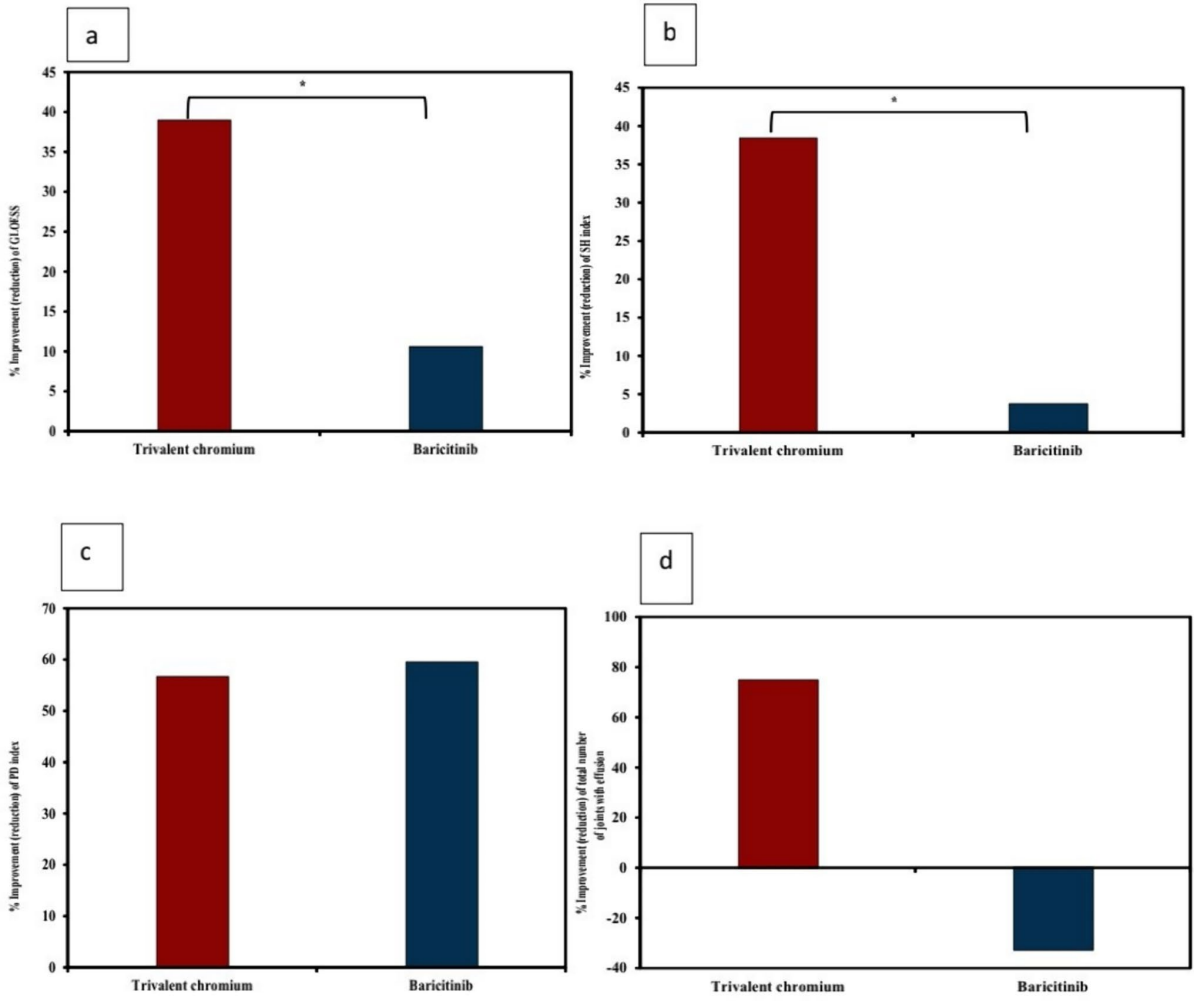
Improvement of hands ultrasound findings **a** improvement of GLOESS in both groups, **b** improvement of SH in both groups, **c** improvement of PDUS in both groups and **d** the decrease (improvement) of number of effusions with Cr III and its deterioration (increase) with baricitinib on the third visit. *: Significant difference

**Fig. 4:**
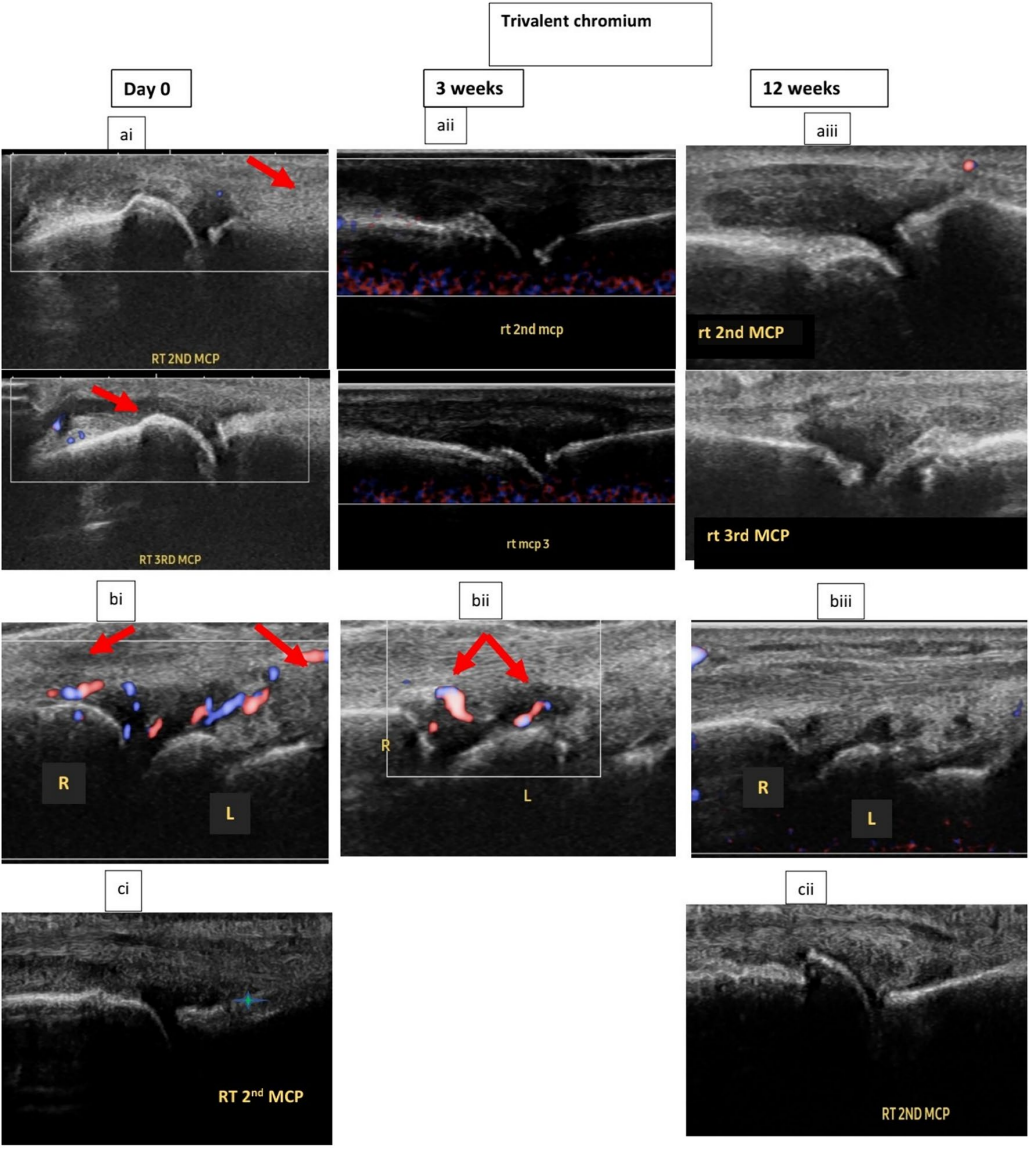
Hands of ultrasound of some patients who received Cr III, **ai** red arrow indicates PDUS and blue sign indicates effusion.**ai** Hands ultrasound of the first patient on the first visit revealed that: GLOESS = 49, SH = 49, PDUS = 3 in the right second, third and fourth metacarpophalangeal (MCP) joints, (red arrows showed in the image indicates PDUS in the second and third MCP joints), number of joints effusions = 16. **aii** Second visit revealed: doppler activity disappeared (PDUS = 0). **aiii** Third visit’s ultrasound showed: GLOESS decreased = 35, SH decreased = 35, PDUS = 0, effusions disappeared (number of effusions = 0). **Bi** Hands ultrasound of the second patient revealed that: **bi** First visit GLOESS: 52, SH = 52, PDUS = 5 (doppler activity was present in left radio-lunate (RL) and intercarpal joints) (red arrow shows PDUS in the left RL joint), number of effusions = 11. **bii** On the second visit, PDUS decreased. **biii** On the third visit, GLOESS decreased = 41, SH decreased = 41, PDUS decreased = 2, number of effusions decreased = 4. **ci** Ultrasound of the first visit showed that: GLOESS = 55, SH = 55, number of joints effusions = 16. **Cii**: Ultrasound of the third visit showed that: GLOESS decreased = 31, SH decreased = 31, number of effusions decreased = 2. (The blue sign shows that effusion in the second MCP **ci** that was present on the first visit disappeared in **cii** on the third visit

**Fig. 5:**
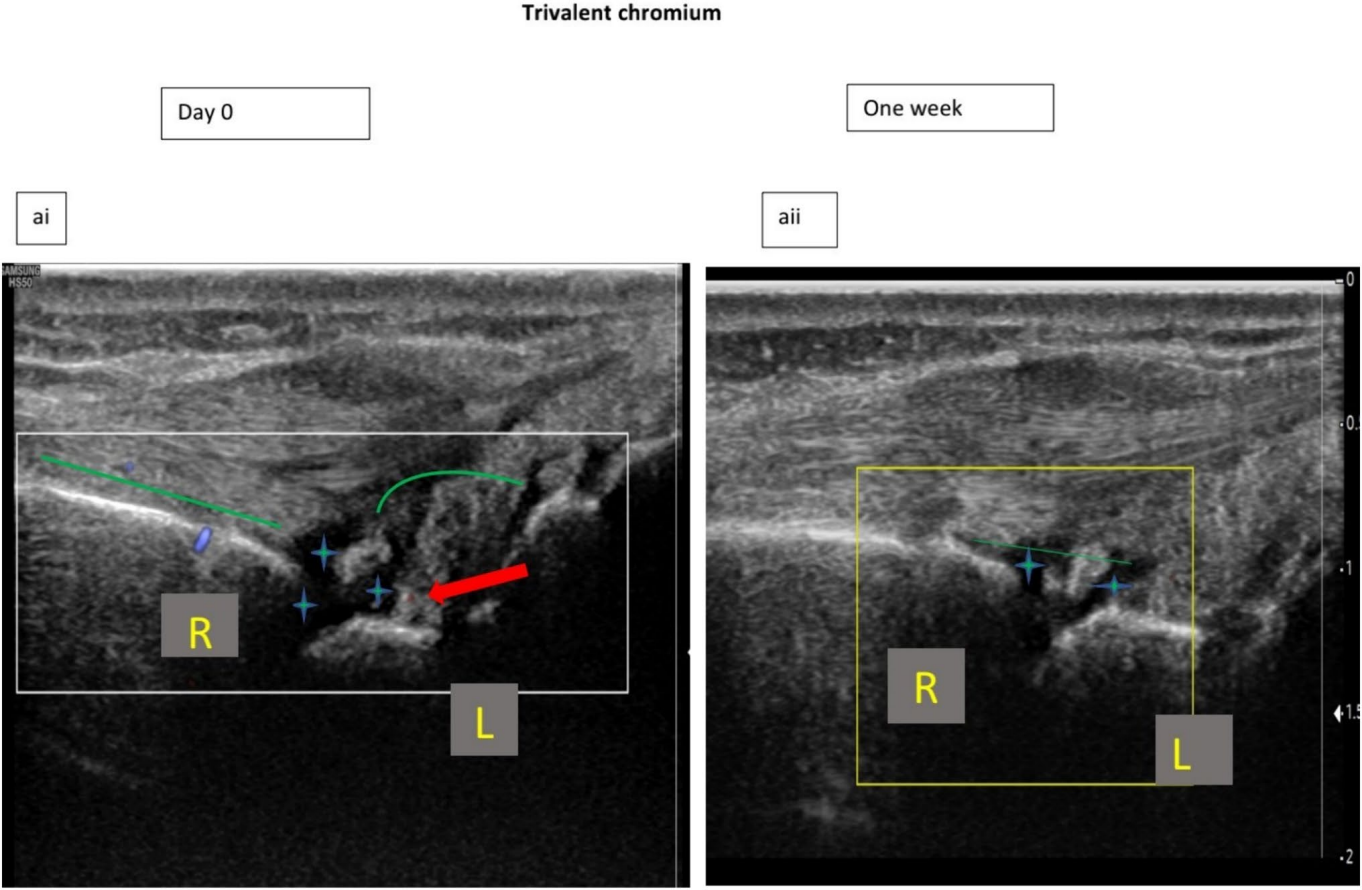
The hands ultrasound of one patient who received Cr III for 1 week, (i)Hands ultrasound in the first visit revealed that : SH : grade 3 (green line), PDUS : grade 2 in the right radio-lunate and left fifth MCP joints (red arrow PDUS = 1 in radio-lunate joint (RL) joint) and blue sign indicates the effusion in the same joint. Ii) after 1 week of Cr III intake, the ultrasound was done on day 11 and showed that: SH decreased to be of grade 2, PDUS in both joints disappeared = 0, effusion decreased

Hands ultrasound findings and their improvements are shown in Tables 1 and 2 and Figs. 3, 4 and 5.

Hands ultrasound of the patient who used Cr III supplement for 1 week showed improvement and disappearance of PDUS which was present on the first visit (Fig. 5).

## Safety

### A. Side effects

Side effects which appeared in both groups are shown in Table 3.

**Table 3:** Side effects which appeared in both groups

Side effects	Trivalent chromium (Cr III)	Baricitinib
Death	0	1
Chest pain	0	2 (regarded as ischemic heart disease and pulmonary embolism)
Gastric perforation	0	1
Palpitation	1 (after two doses: intolerance to the dose)	1
Breathlessness	0	3
Numbness, hyper-thesia	0	1
Loin pain, Polyuria and Dark urine	1 (after three doses)	0
Leg pain	0	2
Superficial thrombophlebitis	0	1
Abdominal pain and Vomiting	1 (appeared when used with torsemide and disappeared after decreasing the dose and when the two medicines were taken apart)	1
Nausea	0	1
Increased risk of infections	0	1
Headache and/ or dizziness	3 (in 2 patients was for few day decreased by rest, eating and disappeared after decreasing the dose)	1
Hair fall	0	1
Mild facial swelling	1	0

### B. Monitoring

There was no significant difference in blood count for both groups at the beginning and the end of the study, also no significant difference in renal or liver functions or RBS for Cr III.

## Discussion

RA being one of the debilitating diseases, many patients are treated with medications which cause deterrent side effects and the treatment response differs from a patient to another these reasons make researches always concerned to search for more options which are potentially safe and effective for disease treatment.

Study design was based on monitoring Cr III and baricitinib introduced to patients either on no previous treatment or in patients who are already on conventional DMARDs providing that these DMARDs have not changed within the period the medicine takes for full efficacy or for its effect to disappear. (Cohen et al. 2024) Non-conjugated form of Cr III was chosen in the study to avoid any confounding effect of a conjugate or side effects that may happen when using the conjugated form.

Ultrasound was done for hands joints because they are considered the most commonly affected in the disease. (Fleming et al. 1976).

Cr III showed great results for RA treatment with symptomatic relief, clinical and sonographic improvement which appeared even on the first few days of its intake, this proves what some studies showed, that Cr III is being an immunomodulatory regulating many pathways and having anti-inflammatory properties (Mohamed 2016), (Hassouna et al. 2022), (Moradi et al. 2019), (Jain et al. 2010), (Hoffman et al. 2014), (Kolahian et al. 2015), (Rhee et al. 2002) and agrees with results of its usage in disease model. (Mohamed 2016).

Study results for baricitinib in RA also agrees with its effects in disease treatment. (Keystone et al. 2015) Baricitinib is recently considered one of the powerful immune-modulatory agents, however, introducing the presumably safer Cr III showed marvelous results in comparison to baricitinib.

Side effects which appeared in the patients’ group taking Cr III were mild to some extent. Going into details; most symptoms that appeared were headache and/or dizziness which then disappeared after decreasing the dose and relieved by eating or rest, only one patient stopped the supplement after disease improvement for complaining of headache and weight loss, however, the patient recommended engagement in the study for another subject. Another temporary side effect was abdominal pain and vomiting which happened to one patient when taken concomitantly with torsemide used by the patient as a hypertensive, when the patient decreased the dose of the supplement and the two medications was taken apart, side effects disappeared after a few days. Intolerance to Cr III supplement happened in two patients after two or three doses, the first patient complained of palpitation, this may be due to mildly elevated liver enzymes before taking the supplement, this may explain why this symptom appeared when blood sugar level may have changed, (Rosen et al. 2016), however, it is important to mention that case reports of irregular beats were associating large dose of Cr III. Web MD (2023).

The second patient; who was intolerant to Cr III; complained of loin pain, polyuria and dark urine, this may have many explanations that it was either an acute kidney injury, allergic interstitial nephritis, just polyuria occurring with heavy metals (O'Brien 2024), (Lentini et al. 2017) or other associating genitourinary condition e.g. infection or stone, etc.… unfortunately the patient refused to attend for further investigations, the latter two patients were both of low body weight which may explain dose intolerance. Only one patient mentioned to have mild facial swelling on the fourth day of taking the supplement, hypersensitivity was reported with Cr III supplementation, Web MD (2023).

This was why it was recommended to stop the medication after 1 week. Despite that disease improvement in this patient was evident clinically and on sonographic assessment in these few days.

On the other hand, side effects in baricitinib group were more serious and even sometimes fatal. Side effects which occurred with baricitinib agrees with literature recording occurrence of insults (U.S. Food and Drug Administration 2018), (U.S. Food and Drug Administration 2022) such as ischemic heart disease, pulmonary embolism, gut perforation which may be explained by hypercoagulability and/ or increased serum lipid associated with the medication use which may raise the possibility of cardiovascular accidents in RA due to increased incidence of atherosclerosis in the disease. (Web MD 2024a) Other manifestations such as gastrointestinal upset, constitutional symptoms found in patients group taking baricitinib are reported with the drug intake. (Carbone et al. 2020).

To sum up, side effects found with Cr III group much milder than those found with baricitinib (European Medicines Agency 2024), (Versus Arthritis 2024), (Medicines and Healthcare products Regulatory Agency 2020) and other DMARDs (Web MD 2024b) and even symptoms that appeared more alarming with Cr III are already seen with baricitinib in previous studies. (European Medicines Agency 2024), (Versus Arthritis 2024), (Medicines and Healthcare products Regulatory Agency 2020) Meanwhile, Cr III harmlessness is still controversial. It is thought to be genotoxic, hence carcinogenicity on long-term use should be excluded, (Cleveland Clinic 2022), (Cai et al. 2018), (Balali-Mood et al. 2021) also being one of the heavy metals; hepatotoxicity and nephrotoxicity on using the supplement for long periods should be tested.

## Recommendations

Eventually, evaluation of Cr III efficacy, safety and possibility of its usage for other autoimmune and autoinflammatory diseases should be settled.

## Data Availability

Available on request.

## Abbreviations

RA: Rheumatoid arthritis
Cr III: Trivalent chromium
TJC: Tender joint count
SJC: Swollen joint count
HAQ-DI: Health assessment questionnaire disability index
CDAI: Clinical disease activity index
SDAI: Simple disease activity index
DAS28-CRP: Disease activity score in 28 joints calculated with CRP
DAS28-ESR: Disease activity score in 28 joints calculated with ESR
ACR response: American college of rheumatology treatment response
GLOESS: Global OMERACT-EULAR Synovitis Score
SH: Synovial hypertrophy
PDUS: Power Doppler/ grey scale in ultrasound
